# A Novel Method for Classifying Body Mass Index on the Basis of Speech Signals for Future Clinical Applications: A Pilot Study

**DOI:** 10.1155/2013/150265

**Published:** 2013-03-14

**Authors:** Bum Ju Lee, Boncho Ku, Jun-Su Jang, Jong Yeol Kim

**Affiliations:** Medical Research Division, Korea Institute of Oriental Medicine, 1672 Yuseongdae-ro, Yuseong-gu, Deajeon 305-811, Republic of Korea

## Abstract

Obesity is a serious public health problem because of the risk factors for diseases and psychological problems. The focus of this study is to diagnose the patient BMI (body mass index) status without weight and height measurements for the use in future clinical applications. In this paper, we first propose a method for classifying the normal and the overweight using only speech signals. Also, we perform a statistical analysis of the features from speech signals. Based on 1830 subjects, the accuracy and AUC (area under the ROC curve) of age- and gender-specific classifications ranged from 60.4 to 73.8% and from 0.628 to 0.738, respectively. We identified several features that were significantly different between normal and overweight subjects (*P* < 0.05). Also, we found compact and discriminatory feature subsets for building models for diagnosing normal or overweight individuals through wrapper-based feature subset selection. Our results showed that predicting BMI status is possible using a combination of speech features, even though significant features are rare and weak in age- and gender-specific groups and that the classification accuracy with feature selection was higher than that without feature selection. Our method has the potential to be used in future clinical applications such as automatic BMI diagnosis in telemedicine or remote healthcare.

## 1. Introduction

Worldwide, increasing numbers of people are becoming obese, including adults, adolescents, and children and both men and woman [1, 2]. Obesity refers to excess adipose tissue caused by genetic determinants, excessive eating, insufficient physical movement, and an inappropriate lifestyle [1, 3, 4]. Obesity and being overweight are serious public health problems; obesity has a direct relationship with physical health and psychological health and is a potential risk factor for many diseases, including cardiovascular diseases, stroke, ischemic heart disease, diabetes, and cancer [2, 5–8]. Therefore, it is important to recognize when patients are overweight or obese, and many studies have been performed about the relationship of obesity, as determined by body mass index (BMI), and disease [4, 6, 7, 9–11]. BMI, proposed by Lambert Adolphe Jacques Quetelet, is a measurement criterion presenting the relationship between body weight and height [3] and a commonly used public health method for classifying underweight, normal, overweight, and obese patients.

On the other hand, research on the association of body shape (weight, height), age, and gender with speech signals has been conducted over a long period in various fields such as speech recognition, security technology, and forensic and medical science, and many studies have suggested a strong or weak relationship between body shape and speech signals [12–28]. Previous analysis of body shape and speech signals has determined that there are differences between normal and obese people in terms of the facial skeleton, the function of the upper airway, and the surrounding structure of the upper airway [12], and that there is a significant association of body shape with vocal tract length [13]. In various vocal features, the fundamental frequency (pitch) of men was associated with measurements of body shape and size such as chest circumference and shoulder-hip ratio [14]. In more detail, Evans et al. suggested that the fundamental frequency in men is an indicator of body configuration based on their findings of a significant association of large body shape with low fundamental frequency and a significantly negative correlation between weight and fundamental frequency [14]. Lass et al. [15, 16] showed a relationship among heights, weights, body surface areas, and fundamental frequencies of a speaker using Pearson correlation coefficients, and they suggested acoustic cues for accurate estimation of speaker height and weight. van Dommelen and Moxness [17] investigated the ability of listeners to determine the weight and height of speakers using average fundamental frequency, energy below 1 kHz, and speech rate. Although they did not find any significant correlations between these features and the height or weight of the speaker, they suggested that speech rate is a good predictor of the weight of male speakers. González [18] examined the relationship between formant frequencies and the height and weight of subjects aged 20 to 30 years in Spain and reported a weak relationship between body size and formant frequencies in adults of the same gender; moreover, the relationship was stronger in women than in men. His results contradicted those of Fitch, who reported a strong correlation between body size and formant dispersions in macaques. Furthermore, Künzel [19] analyzed the relationship between average fundamental frequency and the weight and height of subjects in Wiesbaden, Germany, but found no significant correlations between vocal features and weight or height. Meanwhile, in previous studies of the association of gender, age, and cultural factor with speech signals, Childers and Wu [20] studied the automatic recognition of gender using vocal features and found that the second formant frequency is a slightly better predictor of gender than the fundamental frequency. Bruckert et al. [21] investigated the reliability of vocal features as indicators of a speaker's physical features and found that men with small formant dispersion and low-frequency formants tend to be taller and older and have high testosterone levels. They argue that cultural factors must be considered when determining correlations between speakers' height and weight and vocal features. Similarly, Belin et al. [22] argue that vocal habits, cultural factors, and age and gender differences play important roles in shaping voice quality. In addition, forensic speaker classifications in the domains of dialect, foreign accent, sociolect, age, gender, and medical conditions were well summarized by Jessen [23], who stated that auditory and acoustic analyses are essential works for forensic speaker classification.

In this study, we ask whether it is possible to classify BMI status of patients using only voice information. If it is possible to know a patient's BMI category on the basis of voice data—irrespective of height and weight information—this can be used as alternative or subsidiary information for the diagnosis of normal weight or obesity and for prognosis prediction, under the assumption of circumstances such as remote medical care environments and real-time monitoring services to support general treatment or emergency medical services. For example, BMI values are calculated from weight and height (kg/m^2^). Thus, to get the BMI value of patients or potential patients, weight and height must be measured on the spot. However, these measurements are sometimes not suitable for remote healthcare or u-healthcare supporting general treatment and emergency medical service in real time at remote locations, since 22% of patients do not estimate their own weight within ±5 kg, even though patient self-estimates of weight are better than estimates by residents and nurses in emergency department [29]. In remote medicine for real-time communication in remote locations, many patients do not know their exact weight at the time of diagnosis of BMI because the weight of patients was changed slowly or rapidly over time. We must obtain the maximal clinic data of patients rapidly and often with minimal network or telephone time and communication equipment. Because a great deal of medical information is needed for patient care and prognosis prediction [30, 31], telemedicine or remote healthcare system facilitates the quality and quantity of data collection and integration, communication between patients and healthcare systems, preprocessing to optimize medical treatment, and decision support and modification of medical treatment primarily using telephones, computers, fax, and WCU VC (virtual community program) [32–34]. Also, the technologies have the advantages of health improvement, patient convenience, cost effectiveness, economy of time, data accuracy and permanence, and continuous real-time monitoring of chronic disease [35–37].

Our contributions in this study are as follows: we first propose a method for classifying the normal weight or the high weight using speech signals in age-and gender-specific groups. Our method may apply to the development of advanced and automatic methods for individual BMI diagnosis in telemedicine and u-healthcare and assist in the development of a simpler system for BMI measurement. Also, our suggestion that is possible to support context awareness may provide clues to improve the overall quality of emergency service via automatic support of patient BMI information in remote healthcare systems with limited resources. We find discriminatory and meaningful features for normal and overweight diagnoses via a statistical analysis between BMI and speech features and identify a compact and useful feature subset in accordance with the age-and gender-specific analysis. The results will serve to create a better discriminatory feature set and accurate classification models in this field.

## 2. Materials and Methods

### 2.1. Data Preparation

#### 2.1.1. Data Collection

A total of 1830 people participated in this study. Data was collected from subjects in several hospitals and the Korea Institute of Oriental Medicine in the Republic of Korea. Subjects with any voice-related diseases were excluded from this study. Speech recording configurations were as follows: no resonance; room temperature, 20°C (±5°C); noise intensity, <40 dB; and humidity, 40%  (±5%). Personal computers and an external sound card (Blaster Live 24-bit) to avoid noise from the personal computers were used for initial voice acquisition. GoldWave v5.58 was used to record audio data, and the voice files were saved in the wav format. The distance from the subjects' mouth to the microphone (Sennheiser e-835s microphone) was 4–6 cm.

The recording of the speakers' speech was strictly controlled by a standard operating procedure (SOP). The SOP was established to capture the natural characteristics of the speakers in short recordings. The speakers rested for 1 hour before actual recording to reduce suspense. An operator instructed the speakers regarding the recording content, and the speakers were asked to pronounce words in their normal tone without tension. The operator constantly monitored the speakers' speech and their distance from the microphone while recording. When the speakers could not produce a uniform tone for 5 vowels, their speech was rerecorded until they achieved a certain level of tone uniformity. Each sentence was recorded twice, and the value of each feature was obtained by averaging the values of the 2 recordings for more stable features.

**Figure 1:**
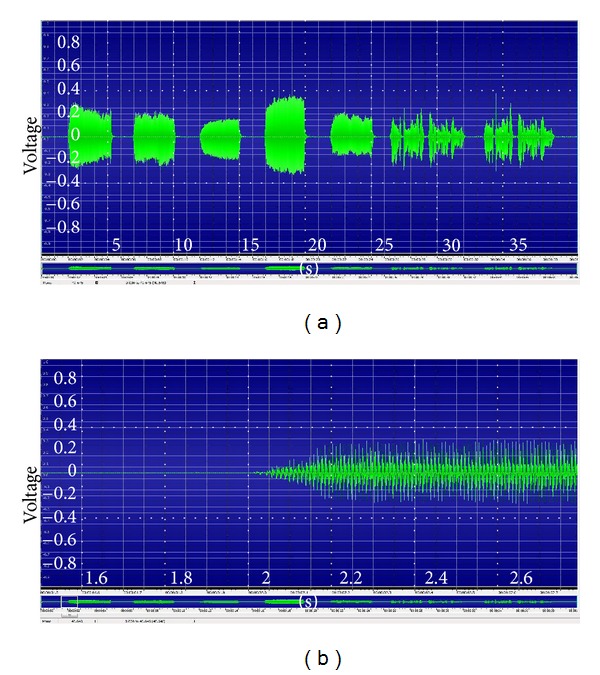
Sample of speech signal recording of 5 vowels and one sentence ((a): signals of 5 vowels and one sentence and (b): detailed signal of one vowel to demonstrate the difference between noise and signal).

All features were extracted using 5 vowels (A, E, I, O, U) and 1 sentence [38]. For speech feature extraction, we extracted 65 features from the collected data set. The extracted features consisted of pitch, average ratio of pitch period, correlation coefficient between F0 and intensity (CORR), absolute Jitter (Jita), and Mel frequency cepstral coefficients (MFCC), among others [18, 23, 27]. The specific content of the extracted features is described in Table 1, and sample of speech signal recording of 5 vowels and one sentence is showed in Figure 1.

#### 2.1.2. Class Label Decision for Normal and Overweight Statuses

Obesity and BMI research is difficult due to different ethnic groups and different national economic statuses [7]. Also, BMI values differ according to physiological factors and environmental factors, such as residing in a city or a rural area. For instance, BMI values of a population in an Asian region tend to be lower than those of a population in a Western region, whereas Asians have risk factors for cardiovascular disease and diabetes related to obesity at relatively low BMI values [9, 39]. The BMI cutoff values for overweight and obesity depend on several factors including ethnicity, rural/urban residence, and economic status [7, 40]. Therefore, we decided that this study's overweight cutoff point of BMI value was ≥23 kg/m^2^, according to suggestions by the World Health Organization and references [39, 41, 42]. We refer here to only 2 classes: the “normal” and the “overweight.” Subjects in the BMI who range from 18.5 to 22.9 were labeled normal, and subjects with a BMI of 23 or over were labeled as overweight. Underweight patients were passed over due the lack of a minimum number of subjects. Finally, we divided the data set into 6 groups for age-and gender-specific classification: female: 20–30 (females aged 20–39 years), female: 40–50 (females aged 40–59 years), female: 60 (females aged 60 years and over), male: 20–30 (males aged 20–39 years), male: 40–50 (males aged 40–59 years), and male: 60 (males aged 60 years and over).

The overall mean ages of the female and male subjects were 41.79 and 40.51, respectively. The mean age and standard deviation of females aged 20–39 years were 28.22 and ±6.326, and the mean BMI and standard deviation were 21.76 and ±2.489. The rest of the groups are described in Table 2. The number of normal and overweight subjects in the 6 groups is described in Table 4.

### 2.2. Feature Selection and Experiment Configurations

For feature subset selection, we applied normalization (scale 0~1 value) to all data sets. The Wrapper-based feature selection approach [43, 44] using machine learning of logistic regression [30, 45] with genetic search was used to maximize the area under ROC curve (AUC). The selected features in each group are shown in Table 3. All experiments were performed using logistic regression in Weka [46], and a 10-fold cross validation was performed [47]. We used the accuracy, true positive rate (sensitivity, TPR), false positive rate (1 specificity, FPR), precision, and F measure as performance evaluation criteria [47, 48]. A large proportion of classification algorithms may not solve the class-size imbalance problem [49]. Thus, the accuracy of many classification experiments is higher for a majority class than for a minority class. Therefore, we also evaluated performance using AUC. An ROC curve (receiver operating characteristic curve) represents the balance of sensitivity versus 1 specificity [50]. Because the AUC is a threshold-independent measure, AUC is a widely used to quantify the quality of a prediction or classification model in medical science, bioinformatics, medicine statistics, and biology [31, 51–53]. An AUC of 1 means a perfect diagnosis model, an AUC of 0.5 is random diagnosis, and an AUC of 0 is a perfectly wrong diagnosis.

## 3. Results and Discussion

Our experiments were divided into two steps. In the first experiment, we conducted classification of normal and overweight classes with six data sets according to age-and gender-specific groups without feature selection. A goal of the experiment was to measure the ability to distinguish the normal and the overweight in each group using full features. Also, we want to identify a more compact and discriminatory feature set for detailed classification of each group. Therefore, in the second step, we applied a feature subset selection method to all data sets used in the first experiment. 12 classification models were built in the first and second steps.

### 3.1. Performance Evaluations

**Figure 2:**
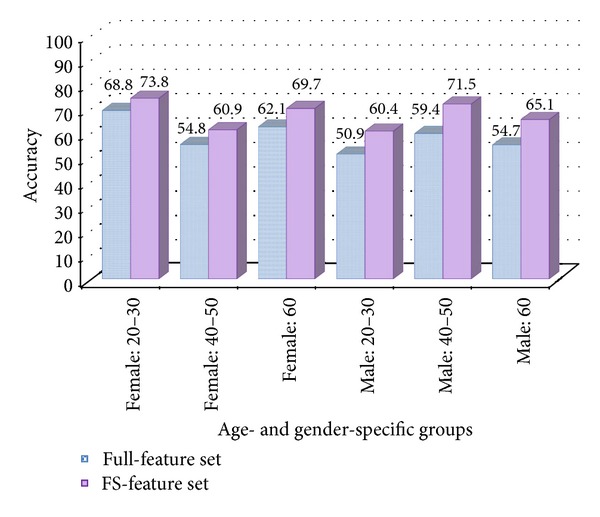
Accuracy comparison of experiment results between full-feature set and FS-feature set in 6 groups.

**Figure 3:**
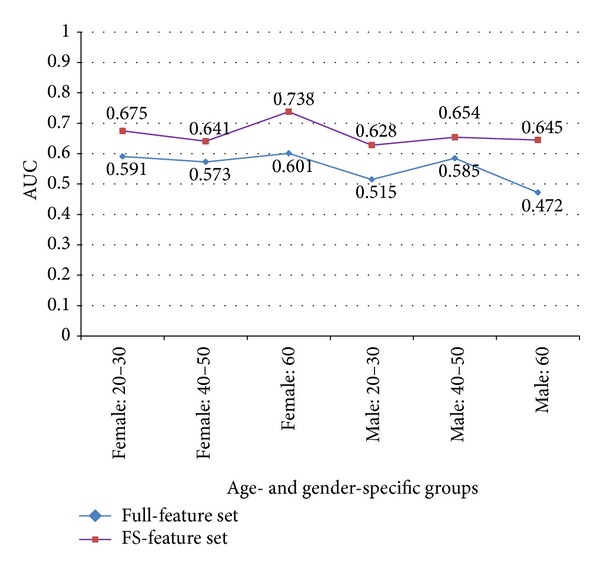
AUC comparison of experiment results between full-feature set and FS-feature set in 6 groups.

All of the performances in experiments applied to feature selection (FS-feature sets) in age- and gender-specific experiments were superior than those in experiments without feature selection (full-feature sets). Figures 2 and 3 show that the improvements in AUC and accuracy offered by feature selection were statistically significant. The accuracies for the 6 groups using full-feature sets ranged from 50.9 to 68.8%. After feature selection, the accuracies for the 6 groups using FS-feature sets ranged from 60.4 to 73.8%, and the average accuracy of the 6 groups improved by about 8.4% compared with the use of full-feature sets. The highest accuracy among the groups was 73.8% (female: 20–30), and the lowest accuracy was 60.4% (male: 20–30).

However, AUC results based on sensitivity and false positive rates (1 specificity) were slightly different from the accuracy results. AUC using FS-feature sets ranged from 0.628 to 0.738. The accuracy of the female: 60 group was lower than that of female: 20–30 and male: 40–50, but the AUC of female: 60 was the highest among the 6 groups. The specific performance results of female: 60 using the FS-feature set included a sensitivity of 0.366, FPR of 0.173, precision of 0.455, and F measure of 0.405 in the normal weight class and a sensitivity of 0.827, FPR of 0.634, precision of 0.768, and F measure of 0.796 in the overweight class. The lowest AUC of 0.628 was observed in the male: 20–30 group. Specific experiment results of all groups are described in Table 4.

The confusion matrix (also called a contingency table) in Table 5 describes more detailed performances of 6 models according to age and gender. For example, the classification model of the female: 20–30 group correctly predicted that 337 of 364 subjects with actual normal weight belonged to the “normal” class and that 30 of 133 subjects with actual overweight belonged to the “overweight” class. Moreover, the female: 40–50 model correctly predicted that 103 of 201 subjects with actual normal weight belonged to the “normal” class and that 168 of 244 subjects with actual overweight belonged to the “overweight” class.

Our experiments show that classification of normal and overweight status in the female: 40–50 and male: 20–30 groups was slightly difficult, compared with the other 4 groups and that classification of normal status and overweight status in the female: 20–30 and female: 60 groups was superior compared with the other groups. The classification performance with wrapper-based feature selection was better than that without feature selection. Many of features selected by feature selection differed according to age- and gender-specific groups (see Table 3).

### 3.2. Statistical Analysis of Features Associated with Normal Weight and Overweight

The statistical data are expressed as mean ± standard deviation. Comparisons between normal and overweight groups were performed using independent two-sample *t*-tests, and the *P* values were adjusted using the Benjamin-Hochberg method to control the false discovery rate; *P* values <0.05 and adjusted *P* values <0.05 were considered statistically significant. Only statistically significant features among all features selected by wrapper-based feature subset selection in each group are described in Table 6. All statistical analyses were conducted using SPSS Statistics 19 and R package 2.15.0 for Windows.

In the female 20–30 group, 7 features were significantly different between the normal and the overweight classes (*P* < 0.05 and adjusted *P* < 0.05). In this group, aF60_120_F240_480, aF240_480_960_1960, aF60_120_960_1960, and eF240_480_960_1960 (features related to the ratios of energies) were significantly different between the 2 classes (*P* < 0.001, adjusted *P* = 0.005; *P* < 0.001, adjusted *P* = 0.005; *P* < 0.001, adjusted *P* = 0.005; and *P* < 0.001, adjusted *P* = 0.01, resp.). These results indicate that the ratios of voice energies over the fixed frequency band in normal subjects are higher than those of the overweight subjects in this group. There were statistically significant differences with respect to eMFCC4 and oMFCC4 between the 2 classes (*P* < 0.05, adjusted *P* < 0.05; and *P* < 0.01, adjusted *P* < 0.05, resp.); particularly, the MFCC4 of vowel E and MFCC4 of vowel O of normal subjects (1.277 ± 6.836 and − 4.087 ± 5.624, resp.) were higher than those of overweight subjects (− 0.801 ± 8.315 and − 5.989 ± 7.191, resp.) in this group. In addition, SITS was significantly different between the 2 classes (*P* < 0.005, adjusted *P* = 0.01). This result indicates that the average intensity of sentences in normal subjects (56.14 ± 7.515) is higher than that of overweight subjects (53.73 ± 8.074) among females aged 20–39 years.

In the male 20–30 group, one eMFCC4 feature was significantly different between the normal and the overweight classes (*P* < 0.001, adjusted *P* < 0.01). The MFCC4 of vowel E in normal subjects was higher than that of overweight subjects in this group. None of the features were significantly different within the other groups.

Despite the high accuracy and AUC of classification in the female ≥60 group, no statistically significant differences were detected between the normal and overweight classes. Furthermore, we did not find features with a broad range of applicability for classifying the normal and overweight statuses in the age-or gender-specific classifications. We will discuss these problems further in Section 3.4.

### 3.3. Scalability and Applications

Some studies on patient BMI and weight estimation have focused on emergency medical services and telemedicine because the precise estimation of weight and BMI status in emergency medical care is very important for accurate counter-shock voltage calculation, drug dosage estimation, intensive care, and elderly trauma management [29, 54–57]. Although some issues must be addressed for accurate prediction of the BMI status, our method may have potential applications in telemedicine, remote healthcare, and real-time monitoring services to monitor the BMI status of patients with long-term obesity-related diseases. Additionally, our method can be applied in the diagnosis of individual constitution types in remote healthcare. Pham et al. suggested that the BMI and cheek-to-jaw width ratio were the most important predictive factors for the TaeEum (TE) constitution type [58], and Chae et al. proposed that the TE type tends to have a higher BMI than other types [59]. Furthermore, several studies mentioned that constitution types differed in speech features and body shape (BMI) [60–62]. Thus, through more studies on voice signals, u-healthcare, body shape, and constitutions, the proposed classification method for BMI can be used to diagnose a constitution for personalized medical care, as the BMI is important in both alternative and Western medicines.

### 3.4. Limitations and Future Work

In our study, voice data of subjects were collected by a recording equipment in hospital site and research center site. In order to apply real-time diagnosis in telemedicine or u-healthcare system, additional and important studies such as noise filter, adjustment technique, and handling of atypical speech in emergency, should be performed because of noise or interference generated by network or equipment during telecommunication.

Our method classified only normal and overweight classes and used voice data collected only from Korea. So, in order to more accurately classify a broad range of classes—such as underweight, normal, overweight, obese 1, obese 2, and obese 3—according to WHO standard classification in various ethnic groups, we must collect more and varied data sets.

In our classification experiments, the AUC with feature selection in the female ≥60 group was the highest among all groups, although there were no significantly different features between the 2 classes among surviving features from the feature subset selection in the female ≥60 group. We consider 2 aspects that could be responsible for the occurrence of this problem. First, this could be due to a combination problem of features in wrapper-based feature subset selection and classification problems. From the perspective of machine learning and data mining, machine learning for wrapper-based feature selection is considered a perfect black box. In general, greater numbers of features exhibiting significant differences lead to better machine-learning performance. However, we cannot guarantee that a classification using only significant features (i.e., those with *P* values <0.05) always performs better than one using a combination of significant and less significant features. Therefore, the most important factor is the selection and combination of the features of each group. For example, Guyon and Elisseeff [43] suggest that the performance of variables that are ineffective by themselves can be improved significantly when combined with others. Furthermore, adding presumably redundant variables can result in noise reduction and consequently better class separation. The other possible reason for the observed problem is the lack of samples, which can force under- or overfitting in machine learning. The small sample size is a critical limitation of this study, because our sample size was not representative of the population. Thus, this study should be designated as a pilot observational study. In order to reduce or understand this problem, we require more samples and are currently collecting more samples.

In the future, we will investigate the extraction of useful features that demonstrate statistical significance in all age-and gender-specific groups, build a more accurate classification model, and collect more data for better classification performance. Furthermore, we will examine the association of the BMI with features such as respiration rate from nonstructured speech signals using a new protocol.

## 4. Conclusions

The classification of normal and overweight according to body mass index (BMI) is only possible through the measurement and calculation of weight and height. This study suggested a novel method for BMI classification by speech signal and showed the possibility of predicting a diagnosis of normal status or overweight status on the basis of voice and machine learning. We found discriminatory feature subsets for diagnosing normal or overweight individuals by feature selection. We proved that several features have a statistically significant difference between normal and overweight classes in the female: 20–30 group and male: 20–30 group through statistical analysis of the features selected by feature selection in each group. Our findings showed the possibility to predict BMI diagnosis using a combination of voice features without additional weight and height measurements, even if significant features are rare and weak. The prediction performance with feature selection was higher than that without feature selection. However, the accuracy and AUC achieved by our classification experiment were not yet sufficient for rigorous diagnosis and medical purposes. Therefore, we need more research about discriminatory features of broad range, rich data, and a more accurate classification model.

## Figures and Tables

**Table 1:** All features used in this study and brief descriptions.

Feature	Brief description	Feature	Brief description
aF0	Basic pitch of A	oPPQ	Smoothing value around JITA of O
aJITA	Mean ratio of change in pitch period of A	oF60_120_240_480	(energy of 60~120 Hz)/(energy of 240~480 Hz) of O
aJITT	Percentage of JITA value of A	oF240_480_960_1920	(energy of 240~480 Hz)/(energy of 960~1920 Hz) of O
aPPQ	Smoothing value around JITA of A	oF60_120_oF960_1920	(energy of 60~120 Hz)/(energy of 960~1920 Hz) of O
aF60_120_F240_480	(energy of 60~120 Hz)/(energy of 240~480 Hz) of A	oF1	Formant of first in 4 frequency periods of O
aF240_480_960_1920	(energy of 240~480 Hz)/(energy of 960~1920 Hz) of A	oF2	Formant of second in 4 frequency periods of O
aF60_120_960_1920	(energy of 60~120 Hz)/(energy of 960~1920 Hz) of A	oF2_F1	Difference of frequencies (oF2-F1)
aF1	Formant of first in 4 frequency periods of A	uF0	Basic pitch of U
aF2	Formant of second in 4 frequency periods of A	uJITA	Mean ratio of change in pitch period of U
aF2_F1	aF2/F1	uJITT	Percentage of JITA value of U
eF0	Basic pitch of E	uPPQ	Smoothing value around JITA of U
eJITA	Mean ratio of change in pitch period of E	uF60_120_240_480	(energy of 60~120 Hz)/(energy of 240~480 Hz) of U
eJITT	Percentage of JITA value of E	uF240_480_960_1920	(energy of 240~480 Hz)/(energy of 960~1920 Hz) of U
ePPQ	Smoothing value around JITA of E	uF60_120_960_1920	(energy of 60~120 Hz)/(energy of 960~1920 Hz) of U
eF60_120_240_480	(energy of 60~120 Hz)/(energy of 240~480 Hz) of E	uF1	Formant of first in 4 frequency periods of U
eF240_480_960_1920	(energy of 240~480 Hz)/(energy of 960~1920 Hz) of E	uF2	Formant of second in 4 frequency periods of U
eF60_120_960_1920	(energy of 60~120 Hz)/(energy of 960~1920 Hz) of E	uF2_F1	Difference of frequencies (uF2-F1)
eF1	Formant of first in 4 frequency periods of E	iF0_aF0	Difference of frequencies (iF0-aF0)
eF2	Formant of second in 4 frequency periods of E	uF0_oF0	Difference of frequencies (uF0-oF0)
eF2_F1	Difference of frequencies (eF2-F1)	aMFCC4	The terms of Mel frequency cepstral coefficients of A
iF0	Basic pitch of I	eMFCC4	The terms of Mel frequency cepstral coefficients of E
iJITA	Mean ratio of change in pitch period of I	iMFCC4	The terms of Mel frequency cepstral coefficients of I
iJITT	Percentage of JITA value of I	oMFCC4	The terms of Mel frequency cepstral coefficients of O
iPPQ	Smoothing value around JITA of I	uMFCC4	The terms of Mel frequency cepstral coefficients of U
iF60_120_240_480	(energy of 60~120 Hz)/(energy of 240~480 Hz) of I	CORR	Pearson correlation coefficients between F0 and intensity
iF240_480_960_1920	(energy of 240~480 Hz)/(energy of 960~1920 Hz) of I	P50	50th percentile of F0
iF60_120_960_1920	(energy of 60~120 Hz)/(energy of 960~1920 Hz) of I	I50	50th percentile of intensity
iF1	Formant of first in 4 frequency periods of I	SF0	Mean pitch of sentence
iF2	Formant of second in 4 frequency periods of I	SSTD	Standard deviation of mean pitch of sentence
iF2_F1	Difference of frequencies (iF2-F1)	SITS	Intensity average
oF0	Basic pitch of O	SISTD	Standard deviation of intensity
oJITA	Mean ratio of change in pitch period of O	SSPD	Time to read one sentence
oJITT	Percentage of JITA value of O	**Total**	**65**

**Table 2:** Mean and standard deviation of age and BMI by each group.

	Female: 20–30	Female: 40–50	Female: 60	Male: 20–30	Male: 40–50	Male: 60
Age	28.22 ± 6.326	48.7 ± 5.555	67.14 ± 5.254	27.34 ± 5.433	49.24 ± 5.257	66.75 ± 4.995
BMI	21.76 ± 2.489	23.76 ± 3.048	24.96 ± 3.042	23.71 ± 2.971	24.67 ± 3.090	23.59 ± 2.3

**Table 3:** Selected features by feature selection in each group (*N*: number of selected features).

Model (group)	*N*	Selected features
Female: 20–30	25	aJITT, aPPQ, aF60_120_F240_480, aF240_480_960_1960, aF60_120_960_1960, aF1, eF0, eJITA, ePPQ, eF240_480_960_1960, eF2, iPPQ, iF60_120_960_1960, oF0, oJITT, oF1, oF2, uF0, uJITT, aMFCC4, eMFCC4, oMFCC4, uMFCC4, SF0, SITS
Female: 40–50	29	aF0, aJITA, aJITT, aF240_480_960_1960, aF2, eF0, eJITT, ePPQ, eF2_F1, iJITA, iPPQ, iF60_120_240_480, iF240_480_960_1960, iF60_120_960_1960, oF0, oF240_480_960_1960, oF1, oF2, uF0, uPPQ, uF60_120_960_1960, uF1, uF2, uF2_F1, aMFCC4, uMFCC4, CORR, I50, SISTD
Female: 60	22	aJITA, aJITT, aF60_120_F240_480, aF240_480_960_1960, eJITT, ePPQ, eF240_480_960_1960, eF2_F1, iF60_120_240_480, iF240_480_960_1960, iF60_120_960_1960, iF2, oF0, oJITT, oF2, oF2_F1, uF0, uJITA, uF60_120_240_480, uF60_120_960_1960, uMFCC4, SISTD
Male: 20–30	8	aJITA, aPPQ, eF2, iF1, oJITT, uPPQ, eMFCC4, uMFCC4
Male: 40–50	24	aF0, eF0, eJITA, eJITT, eF60_120_960_1960, eF1, eF2, eF2_F1, iF0, iJITA, iPPQ, iF60_120_240_480, iF240_480_960_1960, iF2, oJITA, oJITT, oPPQ, oF60_120_oF960_1960, oF1, oF2, uF0, uF60_120_960_1960, eMFCC4, SF0
Male: 60	23	aJITT, aF60_120_F240_480, aF60_120_960_1960, aF1, eF240_480_960_1960, eF1, eF2_F1, iJITA, iPPQ, iF240_480_960_1960, iF60_120_960_1960, iF1, iF2, iF2_F1, oF60_120_240_480, uF2, uF0_oF0, oMFCC4, P50, I50, SSTD, SITS, SSPD

**Table 4:** Specific performance results (with feature selection, *N*: number of subjects of each class).

Model (group)	Class	*N*	Sensitivity	False positive rate (1-specificity)	Precision	*F* Measure
Female: 20–30	Normal	364	0.926	0.774	0.766	0.838
	Overweight	133	0.226	0.074	0.526	0.316
Female: 40–50	Normal	201	0.512	0.311	0.575	0.542
	Overweight	244	0.689	0.488	0.632	0.659
Female: 60	Normal	41	0.366	0.173	0.455	0.405
	Overweight	104	0.827	0.634	0.768	0.796
Male: 20–30	Normal	175	0.52	0.325	0.576	0.547
	Overweight	206	0.675	0.48	0.623	0.648
Male: 40–50	Normal	77	0.377	0.14	0.537	0.443
	Overweight	179	0.86	0.623	0.762	0.808
Male: 60	Normal	35	0.429	0.239	0.469	0.448
	Overweight	71	0.761	0.571	0.73	0.745

**Table 5:** Confusion matrix (also called contingency table or error matrix) of 6 models according to age and gender in classification experiments with feature selection.

		Classification model^a^			
Group	Actual	Predicted		Subjects^b^	
		Overweight	Normal	Overweight	Normal
Female: 20–30	Overweight	30	103	133	364
	Normal	27	337		
Female: 40–50	Overweight	168	76	244	201
	Normal	98	103		
Female: 60	Overweight	86	18	104	41
	Normal	26	15		
Male: 20–30	Overweight	139	67	206	175
	Normal	84	91		
Male: 40–50	Overweight	154	25	179	77
	Normal	48	29		
Male: 60	Overweight	54	17	71	35
	Normal	20	15		

^a^Results of confusion matrix by classification model; ^b^number of subjects of each class (overweight and normal) in original data.

**Table 6:** Statistical analysis results by independent two sample *t*-test and Benjamin-Hochberg's method.

Group	Feature	Class	Mean	Std.	*T*	*P* value	Adj. *P* value
Female: 20–30	aF60_120_F240_480	Normal	0.834	0.390	3.474	<0.001	0.005
		Overweight	0.699	0.365			
	aF240_480_960_1960	Normal	2.285	0.818	3.510	<0.001	0.005
		Overweight	1.996	0.806			
	aF60_120_960_1960	Normal	2.135	1.416	3.618	<0.001	0.005
		Overweight	1.631	1.248			
	eF240_480_960_1960	Normal	3.033	0.627	3.342	<0.001	<0.01
		Overweight	2.818	0.660			
	eMFCC4	Normal	1.277	6.836	2.581	<0.05	<0.05
		Overweight	−0.801	8.315			
	oMFCC4	Normal	−4.087	5.624	2.757	<0.01	<0.05
		Overweight	−5.989	7.191			
	SITS	Normal	56.14	7.515	3.106	<0.005	0.01
		Overweight	53.73	8.074			

Male: 20–30	eMFCC4	Normal	5.057	6.678	3.393	<0.001	<0.01
		Overweight	2.679	6.929			

*P* value < 0.05 was considered statistically significant. The *P* values were adjusted using the Benjamin-Hochberg method to control the false discovery rate. Only statistically significant features among all features selected by wrapper-based feature subset selection in each group are described in this table (Std: standard deviation, Adj: adjusted).
